# Growing divergence between Medicare Advantage plan bids and payments to plans

**DOI:** 10.1093/haschl/qxae093

**Published:** 2024-08-05

**Authors:** Grace McCormack, Erin Trish

**Affiliations:** Price School of Public Policy, Schaeffer Center, University of Southern California, Los Angeles, CA 90089, United States; Price School of Public Policy, Schaeffer Center, University of Southern California, Los Angeles, CA 90089, United States

**Keywords:** Medicare Advantage, Medicare, payment reform, healthcare reform, ACA

## Abstract

As the Medicare Advantage (MA) program grows in enrollment and costs, there has been increasing concern that federal payments to MA plans exceed necessary levels. Estimates suggest that, in 2023, MA plans were paid up to 6% more per enrollee than would have been spent had that beneficiary instead enrolled in traditional Medicare (TM). We evaluated the factors driving this overpayment, characterizing trends in MA benchmarks, bids, and total payments from pre-Affordable Care Act (pre-ACA) levels through 2023. We found that, despite an overall decrease in risk-adjusted bids relative to average risk-adjusted TM enrollee costs, total payments to plans have modestly increased since 2015. Decomposing these trends into various factors in the MA payment formula, we found that divergent trends in benchmarks and bids are, in part, due to the increasing influence of payment adjustments, such as quartile spending adjustments, quality bonus payments, and risk adjustment. Our results suggest that current payment rules have contributed to overpayments and policy reform may be necessary.

## Introduction

As the Medicare Advantage (MA) program grows, there has been increasing concern that federal payments to MA plans exceed necessary levels. For example, the Medicare Payment Advisory Commission (MedPAC) estimates that, in 2023, MA plans were paid up to 6% more per enrollee than would have been spent had that beneficiary instead enrolled in traditional Medicare (TM).^1^

**Figure 1.**
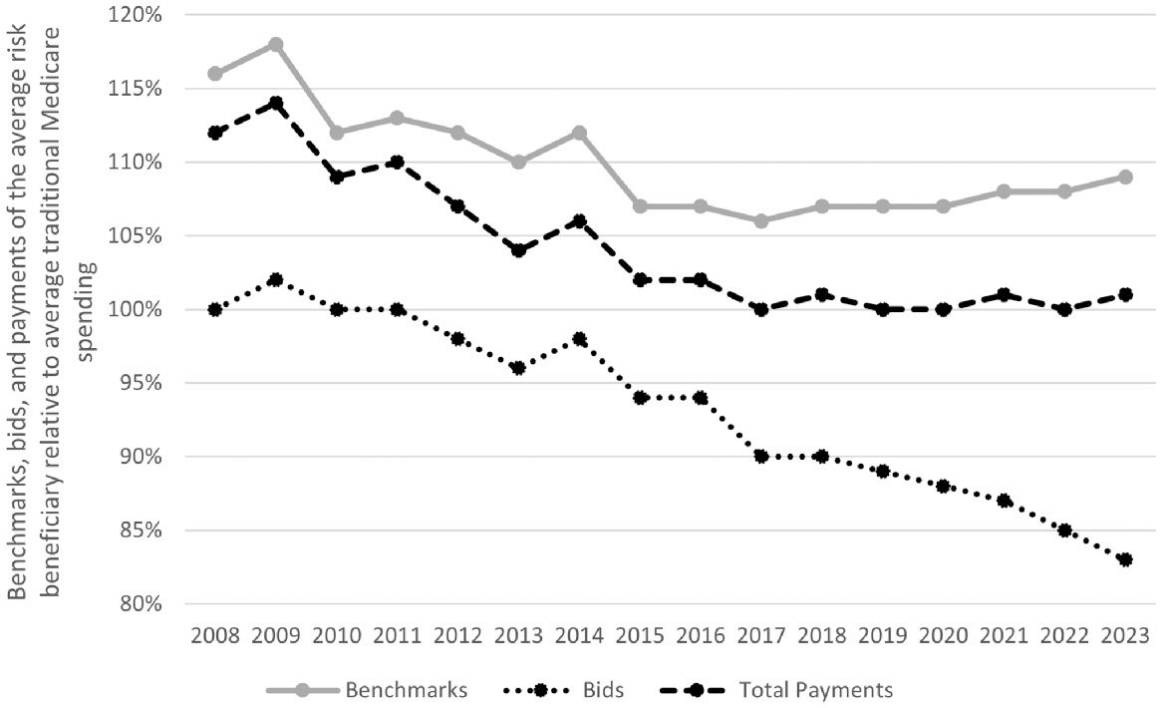
Benchmarks, bids, and payments of the average-risk beneficiary relative to average traditional Medicare (TM) spending.This figure compiles data published annually by the Medicare Payment Advisory Commission (MedPAC). Each line reflects the enrollee-weighted average benchmark, bid, and payments for Medicare Advantage plans in a given year. MedPAC normalizes each metric in 2 steps: first, annual values are normalized by the average risk score in Medicare Advantage so that they reflect the value for an enrollee with a risk score equal to 1, and second, values are normalized by the average risk-adjusted spending of TM enrollees in a given year. For each year, MedPAC uses the previous year's ratebook values and the Centers for Medicare and Medicaid Services (CMS) estimate of spending growth to project TM spending by county. MedPAC further discounts spending related to the double payment for indirect medical education payments made to teaching hospitals. Benchmarks include Quality Bonus Program payments for years, where applicable.

However, since 2010, MA plan bids have decreased considerably, with 2023 MA plan bids averaging 17% less than TM costs (Figure 1). Both benchmarks (the targets against which MA plans bid) and payments to plans (which reflect benchmarks, bids, and other payment formula complexities) declined considerably (relative to TM spending) from 2010 through 2015. Policy changes—including those in the Affordable Care Act (ACA)—likely explain much of the benchmark reductions in the first half of the last decade. Payments to plans fell, reflecting declining benchmarks and plan bids. However, since then, the trends have diverged, with benchmarks and payments to plans holding relatively flat or modestly increasing (relative to TM), in contrast to sustained and considerable reductions in plan bids.

Despite considerable interest in exploring MA payment policy reforms, there is limited research evaluating the factors driving these diverging trends. To better inform these discussions, we evaluated market and policy factors affecting the evolution of payments to MA plans and discuss their implications.

## Medicare Advantage payment policy overview

Medicare Advantage plans bid against county-level benchmarks, the maximum amount the federal government will pay for an MA enrollee. Benchmarks are set by the Centers for Medicare and Medicaid Services (CMS) and reflect TM spending (Parts A and B) for an average-risk beneficiary in that county, with 2 main adjustments: quartile adjustments and Quality Bonus Program (QBP) payments. Quartile adjustments are county-level benchmark adjustments that reduce benchmarks (by up to 5%) in counties with high TM spending and increase benchmarks (by up to 15%) in counties with low TM spending.^2^ QBP payments are contract-level adjustments that increase benchmarks for plans with high-quality ratings (ie, star ratings). The QBP can increase benchmarks by up to 5% in most counties and up to 10% in “double-bonus” counties.^3^ The ACA introduced both the quartile and QBP adjustments in 2012 (alongside other concurrent MA benchmark cuts), phasing in these payment rules until fully implemented in 2017.^4^

Payments to MA plans are determined by comparing plan bids to benchmarks. When a plan bids above the benchmark, the plan receives a monthly payment equal to the benchmark, and enrollees pay the difference through an additional premium.^5^ However, most plans bid below the benchmark. In this scenario, the plan receives a monthly payment equal to its bid, plus an additional rebate. The rebate—which plans must return to enrollees through lower premiums, cost-sharing, and/or supplemental benefits—is calculated as 50%–70% of the difference between the bid and benchmark (with higher percentages for plans receiving higher quality ratings).

Both baseline payments and rebates are risk-adjusted to reflect the health status of a plan's enrollees using the CMS–hierarchical condition category model, incentivizing plans to document conditions more aggressively than in TM.^6^

Together, these policies imply that benchmarks—and thus payments to plans—reflect not only TM spending and plan bids but also additional factors including the plan's quality rating, the county's relative level of TM spending, and the predicted risk of plan enrollees. In the following analyses, we characterize how these various factors impact trends in payments to MA plans.

## Data and methods

For our analysis of benchmarks, bids, and payments relative to TM costs, we used average, normalized data published annually by MedPAC. These data reflect the average benchmarks (including quartile and QBP adjustments), bids, and payments (including rebates) for an MA enrollee with a risk score of 1 compared with the average projected expenditure of a TM enrollee with a risk score of 1. Importantly, these numbers do not adjust for differences in coding intensity or selection beyond that accounted for through risk adjustment and CMS coding intensity adjustments. (See the Supplemental Appendix for details. To access the appendix, click on the Details tab of the article online.)

For our analysis of nominal benchmarks, bids, and payments (ie, absolute rather than relative to TM), we used Plan Payment and Plan Benefits Package files from 2010 to 2021 matched to enrollment and benchmark data from the CMS Landscape and ratebook files. For the period from 2012–2017 as ACA payment rules were phased in, ratebook benchmarks reflect the appropriate weighted pre- and post-ACA benchmarks. Following previous work, we calculated de facto QBP adjustments and double-bonus–eligible counties using the Plan Performance Data and the CMS ratebook.^3^ Risk-adjusted benchmarks are calculated using average plan risk scores from the Plan Payment files. Total governmental payments to a plan are calculated as bids up to the benchmark amount plus rebates times the plan's average risk score. Details and links to data source are available in the Supplemental Appendix.

We restricted to Health Maintenance Organization (HMO)/HMO–Point of Service (HMO-POS), and local Preferred Provider Organization (PPO) plans, excluding Special Needs Plans (SNPs) and Employer Group Health Plans (EGHPs), as well as enrollees residing in Puerto Rico and other territories. In supplemental analyses, we conducted repeated cross-sectional regressions of average plan-level benchmarks on average TM spending per county and present a time series of the coefficient of determination (*R*^2^), characterizing within each year the proportion of benchmark variation that is explained by TM spending.

## Results

Benchmarks, bids, and payments relative to TM spending started to decrease beginning in 2010 (Figure 1). All 3 metrics continued to decline for several years, followed by flattening or modestly increasing payments relative to TM starting around 2018 despite persistent declines in plan bids. Average benchmarks have persistently remained higher than TM spending.

**Figure 2.**
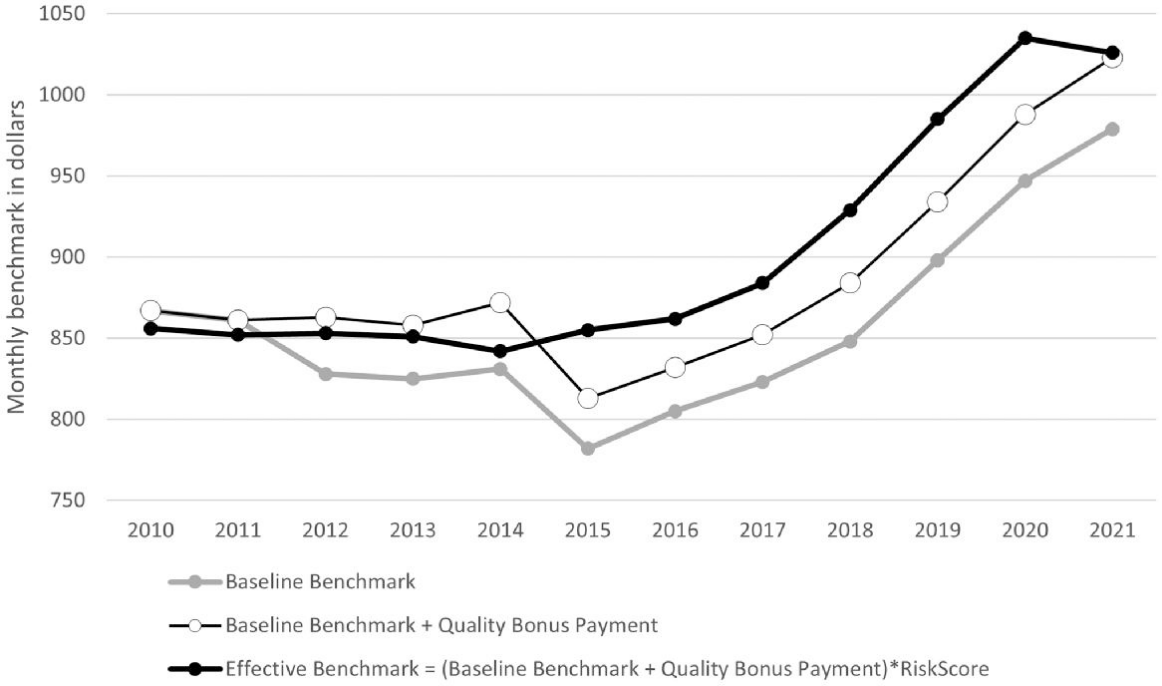
Monthly benchmark time series.This figure shows the average monthly benchmarks for Medicare Advantage plans in each year. The baseline benchmark reflects the baseline benchmark prior to any Quality Bonus Program (QBP) payments or risk score adjustments. This baseline benchmark thus reflects the average cost of county traditional Medicare enrollees, adjusted for the traditional Medicare spending quartile. QBP payments are incorporated into benchmark payments starting in 2012. Finally, the effective benchmark reflects the total benchmark including QBP, multiplied by the average plan enrollee risk score. Metrics are enrollee-weighted. This figure restricts to non–Special Needs Plans (non-SNPs), non–Employer Group Health Plans (non-EGHPs) that are either Health Maintenance Organization (HMO) or local Preferred Provider Organization (PPO) plans.

**Figure 3.**
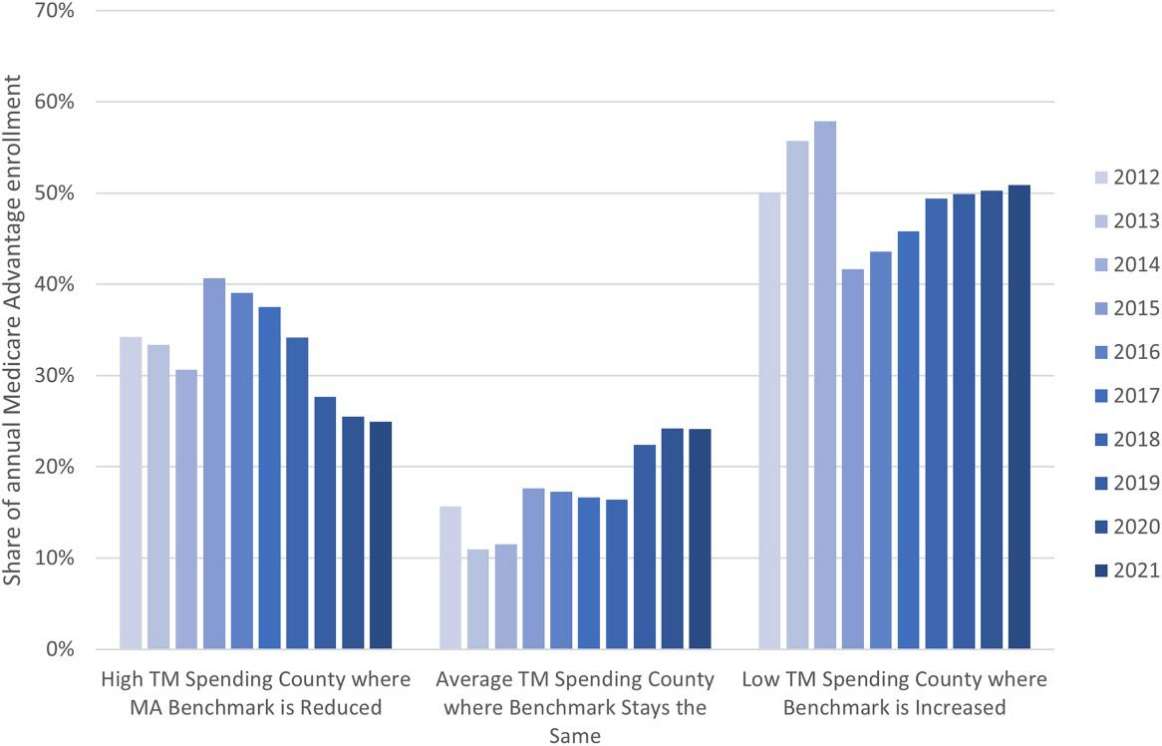
Share of total annual enrollment by county quartile category (ie, benchmark decreased, stayed the same, or increased).This figure depicts the share of total Medicare Advantage (MA) annual enrollment in each year that is in counties with different levels of quartile adjustments. Moving from left to right, the figure shows the share of enrollees in plans in each year that have benchmarks reduced by quartile adjustments (due to being in a high traditional Medicare [TM] spending county), the share of enrollees in plans that have unadjusted benchmarks (due to being in an average TM spending county), and the share of enrollees in plans that have an increased benchmark (due to being in a low TM spending county). This figure restricts to non–Special Needs Plans (non-SNPs), non–Employer Group Health Plans (non-EGHPs) that are either Health Maintenance Organization (HMO) or local Preferred Provider Organization (PPO) plans.

Baseline benchmarks (ie, not relative to TM spending and prior to QBP payments or risk adjustment) declined from 2010 through 2015, after which they increased annually through 2021 (Figure 2, solid grey line). From 2015 to 2021, an increasing share of MA enrollment was in counties with low TM spending that received upward quartile adjustments (Figure 3). Between 2015 and 2021, the percentage of MA enrollment residing in a county with upward quartile adjustments increased from 42% to over 50%. The enrollment-weighted average quartile benchmark adjustment increased from 2.4% to 4.5% over this time (Appendix FigureA1). Given past work suggesting that this shift in enrollment was not caused by quartile adjustments, their presence likely mitigated potential reductions to benchmarks that would have occurred under similar enrollment patterns.^2^

**Figure 4.**
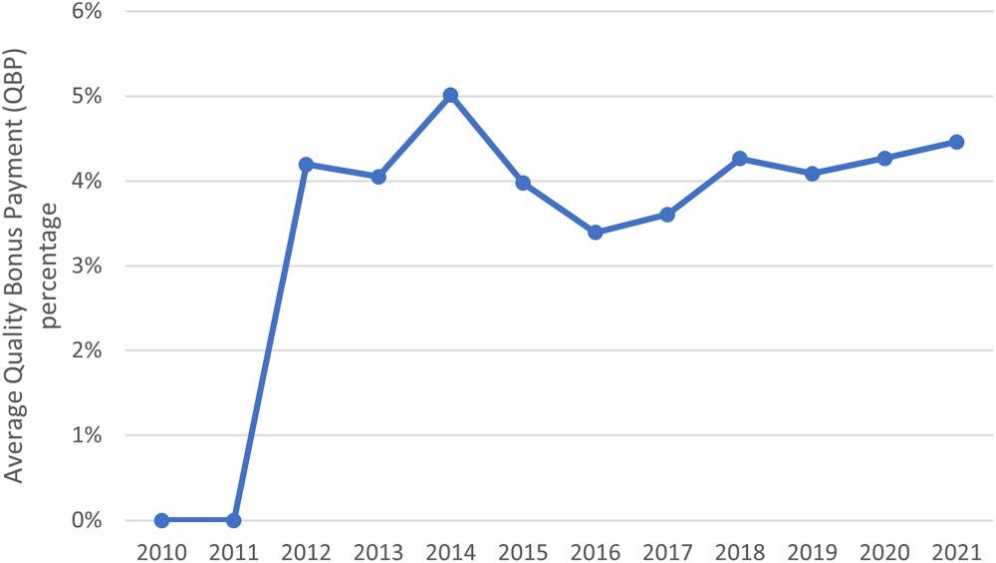
Average per-enrollee de facto Quality Bonus Payment (QBP) percentage time series.This figure shows the average, enrollee-weighted, QBP percentage paid to Medicare Advantage plans each year. The QBP was introduced in 2012. Benchmarks were increased by this QBP off of the baseline benchmark available in their area. That is, in 2021, relative to the baseline benchmark, the average plan received an additional 4.5% in benchmark payments. Metrics are enrollee-weighted. This figure restricts to non–Special Needs Plans (non-SNPs), non–Employer Group Health Plans (non-EGHPs) that are either Health Maintenance Organization (HMO) or local Preferred Provider Organization (PPO) plans.

The introduction of QBP in 2012 further increased average benchmarks relative to baseline as seen by the gap between the grey line and the black and white line in Figure 2. After accounting for double-bonus eligibility and other adjustments, the average enrollee between 2012 and 2021 was consistently in a plan receiving between 3% and 5% QBP bonuses (Figure 4), corresponding to $27 to $44 in monthly payments prior to risk adjustment (Appendix Figure A2). By 2021, 81.9% of MA enrollment was in a plan that was eligible to receive a 5% QBP benchmark increase prior to double bonuses (Appendix Figure A3) and 27% of enrollment was in a double-bonus county (Appendix Figure A4). An increase in the average risk score of MA enrollees in 2015—as well as sustained enrollment of beneficiaries with higher risk scores through 2020 (Appendix Figure A5)—further increased effective benchmarks, as seen by the gap between the red and green lines in Figure 2.

Consistent with the increasing influence of quartile adjustments, QBP adjustments, and risk adjustment, effective benchmarks and average TM spending are decreasingly related over time. The portion of variation in effective benchmarks that is explained by variation in TM spending has decreased, especially since 2015 (Appendix Figure A6).

**Figure 5.**
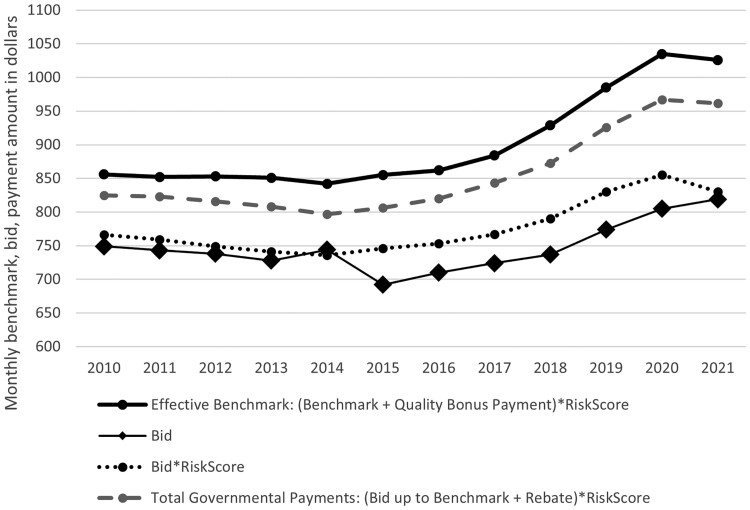
Average per-enrollee monthly bid time series.This figure shows the average effective monthly benchmark, unadjusted plan bid, plan bid adjusted for risk score, and total governmental payments to plans equal to the risk-adjusted plan bid (up to the benchmark) and rebate. Metrics are enrollee-weighted. This figure restricts to non–Special Needs Plans (non-SNPs), non–Employer Group Health Plans (non-EGHPs) that are either Health Maintenance Organization (HMO) or local Preferred Provider Organization (PPO) plans.

**Figure 6.**
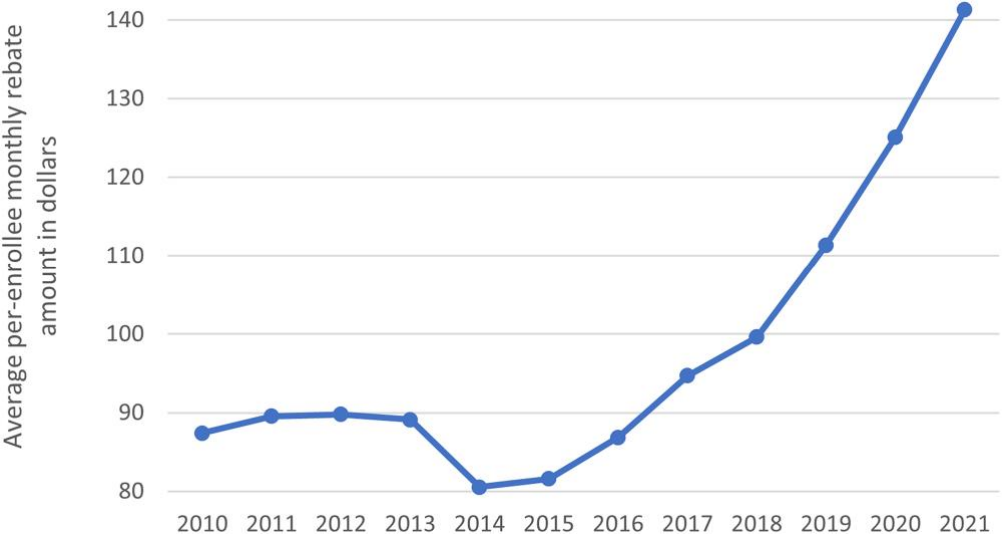
Average per-enrollee monthly rebates time series.This figure shows the average per-enrollee monthly rebate payment in each year. Metrics are enrollee-weighted. This figure restricts to non–Special Needs Plans (non-SNPs), non–Employer Group Health Plans (non-EGHPs) that are either Health Maintenance Organization (HMO) or local Preferred Provider Organization (PPO) plans.

Turning next to plan bids, we found that, while risk-adjusted plan bids increased from 2015 through 2021, these bids grew more slowly than effective benchmarks (Figure 5). As a result, average monthly rebates grew over this time from $82 per enrollee per month in 2015 to $142 in 2021 (Figure 6). In addition to the growing differential between plan bids and benchmarks, rebate growth since 2015 also reflects increases in the percentage of the difference between the bid and benchmark returned to plans via rebates due to higher quality ratings (Appendix Figure A7) and higher average MA risk scores (Appendix Figure A5), since rebates are risk adjusted.

## Discussion

This analysis of trends in benchmarks, bids, and plan payments offers several key insights. First, consistent with prior work,^7^ ACA policy changes led to reductions in benchmarks and payments to plans over the first half of the last decade. However, since then, risk-adjusted benchmarks and payments to plans have leveled off, despite considerable and sustained reductions in plan bids relative to TM.

Second, effective MA benchmarks are high relative to TM spending. Consistent with past work,^1,2,8^ we found that, after incorporating quartile adjustments, QBP payments, and risk adjustment, plans can bid below the benchmark and still receive payments higher than what CMS pays for the average TM enrollee.

Third, MA bids are increasingly lower than both TM spending and effective benchmarks. However, current MA payment policy dictates that, rather than considerably reducing payment to MA plans, much of this difference instead results in increasing rebates, which, in turn, fund premium and cost-sharing reductions and supplemental benefits.

These results are salient for policymakers concerned with payment levels to MA plans. While plans may be paid more for an enrollee than what they would cost CMS had they enrolled in TM, our findings suggest that this dynamic is at least partially the result of payment policy itself, which has resulted in high benchmarks and increasing divergence between plan bids and payments. Under the current MA payment policy framework, increasingly low bids from MA plans generate increasingly generous rebates and supplemental benefits rather than reducing payments to MA plans.^9^ While it is ultimately up to policymakers to debate how much of the medical cost savings generated by MA plans ought to be returned to MA enrollees through benefits rather than to taxpayers, our findings suggest that a careful evaluation of MA payment policy is warranted.

More research is also needed to predict the impact of potential MA payment policy changes. For example, the extent to which current MA payment policy has created the pattern of considerable decreases in plan bids is unclear, as is how plan bids might change under alternative policies. Moreover, the potential impact of payment policy changes on enrollment and benefit generosity for beneficiaries warrants further study. Nonetheless, our findings suggest that—perhaps unsurprisingly—payment policy itself is a key driver of the MA payment levels observed and hotly debated today.

## Supplementary Material

qxae093_Supplementary_Data

## References

[1] Medicare Payment Advisory Commission (US) . Report to the Congress, Medicare Payment Policy. Medicare Payment Advisory Commission; March 2023.

[2] Murray RC , Meyers DJ, Brown EC, Williams TC, Ryan AM. Association between the Medicare Advantage quartile adjustment system and plan behavior and enrollment. JAMA Health Forum. 2024;5(1):e234822. 38214920 10.1001/jamahealthforum.2023.4822 PMC10787313

[3] Layton TJ , Ryan AM. Higher incentive payments in Medicare Advantage's pay-for-performance program did not improve quality but did increase plan offerings. Health Serv Res. 2015;50(6):1810–1828. 26549194 10.1111/1475-6773.12409 PMC4693840

[4] McGuire TG , Newhouse JP, Sinaiko AD. An economic history of Medicare Part C. Milbank Q. 2011;89(2):289–332. 21676024 10.1111/j.1468-0009.2011.00629.x PMC3117270

[5] Medicare Payment Advisory Commission (US) . Medicare Advantage Program Payment System. Medicare Payment Advisory Commission; November 2021.

[6] Geruso M , Layton T. Upcoding: evidence from Medicare on squishy risk adjustment. J Polit Econ. 2020;128(3):984–1026. 10.1086/704756 PMC7384673 32719571

[7] Schwartz AL , Kim S, Navathe AS, Gupta A. Growth of Medicare Advantage after plan payment reductions. JAMA Health Forum. 2023;4(6):e231744. 37354538 10.1001/jamahealthforum.2023.1744 PMC10290750

[8] Ryan AM , Chopra Z, Meyers DJ, Fuse Brown EC, Murray RC, Williams TC. Favorable selection in Medicare Advantage is linked to inflated benchmarks and billions in overpayments to plans: study examines Medicare Advantage favorable selection, benchmarks, and payments to plans. Health Aff (Millwood). 2023;42(9):1190–1197. 37669498 10.1377/hlthaff.2022.01525

[9] McCormack G , Trish E. Trends in the level and composition of supplemental benefits in Medicare Advantage. Health Aff Scholar. 2023;1(1):qxad019. 10.1093/haschl/qxad019 PMC10986227 38756831

