# Supplementary material for: Growing divergence between Medicare Advantage plan bids and payments to plans
Source: Health Aff Sch. 2024 Aug 5;2(8):qxae093. doi: 10.1093/haschl/qxae093 (PMC11342956; doi:10.1093/haschl/qxae093)
Supplement: qxae093_Supplementary_Data [file qxae093_supplementary_data.zip › appendix_revised_v2.docx]

**Supplemental Appendix**

This appendix provides additional detail on the construction of our analysis variables as well as additional results. Section 1 provides additional details on the MedPAC data used for the main text exhibit 1. Section 2 details our construction of variables for the other main text exhibits. Finally, section 3 provides additional results.

**Appendix 1: MedPAC data**

Figure 1 in the main text presents a timeseries of risk-normalized benchmarks, bids, and total payments (bids plus rebates). We construct the underlying data for this table using multiple years of MedPAC reports. These data restrict to non-SNP and non-EGHP plans in each year, though there are small methodological differences exist from year to year with the inclusion of quality bonuses and other changes to payment policy. Below, we document the source of each year of data within the MedPAC reports and the methodological notes that accompany each data report.

| Year | Location | Caption and Additional Notes |
| --- | --- | --- |
| 2010 | March 2010 MedPAC Report to Congress  P.266 Table 4-3 | Benchmarks are the maximum Medicare program payments for MA plans. FFS spending by county is estimated using the 2010 MA rate book. Spending related to the double payment for indirect medical education payments made to teaching hospitals was removed. Totals may not sum due to rounding.  *SNPs and employer-group plans have restricted availability and their enrollment is included in the statistics by plan type. They are presented separately to provide a more complete picture of the MA program. |
| 2011 | March 2011 MedPAC Report to Congress  P.293 Table 12-3 | Benchmarks are the maximum Medicare program payments for MA plans. FFS spending by county is estimated using the 2010 MA rate book. Spending related to the double payment for indirect medical education payments made to teaching hospitals was removed. Totals may not sum due to rounding.  *SNPs and employer-group plans have restricted availability and their enrollment is included in the statistics by plan type. They are presented separately to provide a more complete picture of the MA program. |
| 2012 | March 2012 MedPAC Report to Congress  P.319 Table 12-3 | Benchmarks are the maximum Medicare program payments for MA plans. FFS spending by county is estimated using the 2010 MA rate book. Spending related to the double payment for indirect medical education payments made to teaching hospitals was removed.  *SNPs and employer group plans have restricted availability and their enrollment is included in the statistics by plan type. They are presented separately to provide a more complete picture of the MA program |
| 2013 | March 2013 MedPAC Report to Congress  P.295 Table 13-3 | Benchmarks are the maximum Medicare program payments for MA plans. We estimate FFS spending by county using the 2013 MA rate book. We removed spending related to the remaining double payment for indirect medical education payments made to teaching hospitals. SNPs and employer group plans have restricted availability and their enrollment is included in the statistics by plan type. We have broken them out separately to  provide a more complete picture of the MA program. |
| 2014 | March 2014 MedPAC Report to Congress  P.331 Table 13-3 | Benchmarks are the maximum Medicare program payments for MA plans and incorporate plan quality bonuses. We estimate FFS spending by county using the 2014 MA rate book. We removed spending related to the remaining double payment for indirect medical education payments made to teaching hospitals.  * Benchmarks include both statutory and demonstration bonuses.  ** SNPs and employer group plans have restricted availability, and their enrollment is included in the statistics by plan type. We have broken them out separately to provide a more complete picture of the MA program. |
| 2015 | March 2015 MedPAC Report to Congress  P.325 Table 13-4 | FFS (fee-for-service), MA (Medicare Advantage), PPO (preferred provider organization), PFFS (private fee-for-service), SNP (special needs plan). Benchmarks are the maximum Medicare program payments for MA plans and incorporate plan quality bonuses. We estimate FFS spending by county using the 2015 MA rate book. We removed spending related to the remaining double payment for indirect medical education payments made to teaching hospitals.  * Benchmarks include quality bonuses.  ** SNPs and employer group plans have restricted availability, and their enrollment is included in the statistics by plan type. We have broken them out separately  to provide a more complete picture of the MA program. |
| 2016 | March 2016 MedPAC Report to Congress  P.337 Table 12-4 | We estimate FFS spending by county using the 2016 MA rate book. We removed spending related to the remaining double payment for indirect medical education payments made to teaching hospitals.  * Benchmarks include quality bonuses.  ** SNPs and employer group plans have restricted availability, and their enrollment is included in the statistics by plan type. We have broken them out separately  to provide a more complete picture of the MA program. |
| 2017 | March 2017 MedPAC Report to Congress  P.356 Table 13-6 | Benchmarks are the maximum Medicare program payments for MA plans and incorporate plan quality bonuses. We estimate FFS spending by county using the 2017 MA rate book. We removed spending related to the remaining double payment for indirect medical education payments made to teaching hospitals.  *All numbers in this table have been risk adjusted and reflect quality bonuses, but they have not been adjusted for coding intensity differences between MA and FFS  that exceed the statutory minimum adjustment. |
| 2018 | March 2018 MedPAC Report to Congress  P.364 Table 13-4 | Benchmarks are the maximum Medicare program payments for MA plans and incorporate plan quality bonuses. We estimate FFS spending by county using the 2018 MA rate book.  We removed spending related to the remaining double payment for indirect medical education payments made to teaching hospitals.  *All numbers in this table have been risk adjusted and reflect quality bonuses, but they have not been adjusted for coding intensity differences between MA and FFS  that exceed the statutory minimum adjustment. |
| 2019 | March 2019 MedPAC Report to Congress  P.354 Table 13-3 | Benchmarks are the maximum Medicare program payments for MA plans and incorporate plan quality bonuses. We estimate FFS spending by county using the 2019 MA rate book. We removed spending related to the remaining double payment for indirect medical education payments made to teaching hospitals.  *All numbers in this table have been risk adjusted and reflect quality bonuses, but they have not been adjusted for coding intensity differences between MA and FFS  that exceed the statutory minimum adjustment. |
| 2020 | March 2020 MedPAC Report to Congress  P.376 Table 13-3 | Benchmarks are the maximum Medicare program payments for MA plans and incorporate plan quality bonuses. We estimate FFS spending by county using the 2020 MA rate book. We removed spending related to the remaining double payment for indirect medical education payments made to teaching hospitals.  *All numbers in this table have been risk adjusted and reflect quality bonuses, but they have not been adjusted for coding intensity differences between MA and FFS  that exceed the statutory minimum adjustment. |
| 2021 | March 2021 MedPAC Report to Congress  P.368 Table 12-5 | Benchmarks are the maximum Medicare program payments for MA plans and incorporate plan quality bonuses. We estimate FFS spending by county using the 2021 MA rate book. We removed spending related to the remaining double payment for indirect medical education payments made to teaching hospitals.  The estimate of regional PPO benchmarks relative to FFS corrects the methodology from prior years that used an imputed benchmark amount rather than the benchmark in plan bid data. This correction has no effect on bids or payments for regional PPOs and has no substantive effect on overall benchmark estimates relative to FFS.  The FFS spending denominator used in the table includes all Part A and Part B spending. MA enrollees must be enrolled in both Part A and Part B. For 2017, the Commission estimated that FFS spending for enrollees with both Part A and B was about 1 percent higher than spending for all FFS enrollees. Comparing benchmarks, bids, and payments with spending for FFS enrollees with both Part A and Part B would decrease the overall values for all MA plans in the table by about 1 percentage point.  *All numbers in this table have been risk adjusted and reflect quality bonuses, but they have not been adjusted for coding intensity differences between MA and FFS  that exceed the statutory minimum adjustment.  Our estimates of plan payments do not take into account the impact of the coronavirus pandemic, but given the prospective nature of MA payments, we do not anticipate the pandemic having a substantial impact on MA payments in 2021. We use CMS’s estimate of 2021 fee-for-service (FFS) spending, which uses data through 2019 as the basis for 2021 MA benchmarks, bids, and payments. This estimate also represents the FFS spending levels assumed by plans  when they submitted bids for 2021 in June of 2020. |

**Appendix 2: Public data variable creation**

- **Figure 2**: This figure presents average enrollee-weighted benchmarks in each year. The blue line presents the unadjusted baseline benchmarks. This accounts for quartile adjustments but does not include quality bonus payments and is normalized to reflect amounts for an enrollee with a risk score equal to one. The red line presents the unadjusted baseline benchmarks plus quality bonus payments. Finally, the green line presents the effective benchmark – the benchmark plus quality bonus payments times the average plan risk score. Data for these figures are derived from the following sources:
  - Baseline Benchmarks: We use [ratebook rate calculation data](https://www.cms.gov/medicare/payment/medicare-advantage-rates-statistics/ratebooks-supporting-data), including statutory benchmark data. After the implementation of quality bonus payments, we use the 0% bonus rate as the baseline rate. This rate reflects the rate to county plans, incorporating quartile adjustments and the ACA payment phase-in rate (i.e., the degree to which a county’s benchmarks are reflective of pre- or post-ACA rules. This phase-in took place between 2012 and 2017 so that post-2017 benchmarks fully reflect ACA payment policy).
  - Quality Bonus Payments: After the implementation of quality bonus payments, we use the quality bonus payment benchmarks as reported in the [same [ratebook rate calculation data](https://www.cms.gov/medicare/payment/medicare-advantage-rates-statistics/ratebooks-supporting-data)](https://www.cms.gov/medicare/payment/medicare-advantage-rates-statistics/ratebooks-supporting-data) that we use for the baseline benchmarks. To assign the correct quality bonus payments to each plan, we use the star rating as reported in the [plan performance files](https://www.cms.gov/medicare/health-drug-plans/part-c-d-performance-data) in conjunction with publicly [available](https://www.cms.gov/medicare/health-plans/medicareadvtgspecratestats/downloads/announcement2014.pdf) *Medicare Advantage Capitation Rates and Medicare Advantage and Part D Payment Policies and Final Call Letter* published in each year, which reports the QBP percentage and rebate rate garnered by different star ratings.
  - Risk Scores: We use the publicly published risk scores in the [plan payment data](https://www.cms.gov/medicare/health-drug-plans/plan-payment-data).

All lines are enrollee-weighted derived from the [plan-level enrollment data](https://www.cms.gov/data-research/statistics-trends-and-reports/medicare-advantagepart-d-contract-and-enrollment-data) in the Medicare Advantage (MA) landscape files

- **Figure 3:** This figure presents the share of enrollees in each year who are enrolled in a plan with a given quartile adjustment. [Plan-level enrollment data](https://www.cms.gov/data-research/statistics-trends-and-reports/medicare-advantagepart-d-contract-and-enrollment-data) is derived from the MA landscape files. We assign quartiles using the MA [ratebook rate calculation data](https://www.cms.gov/medicare/payment/medicare-advantage-rates-statistics/ratebooks-supporting-data).
- **Figure 4:** This figure presents the enrollee-weighted quality bonus payment percentages over time. [Plan-level enrollment data](https://www.cms.gov/data-research/statistics-trends-and-reports/medicare-advantagepart-d-contract-and-enrollment-data) is derived from the MA landscape files. We use the star rating as reported in the [plan performance files](https://www.cms.gov/medicare/health-drug-plans/part-c-d-performance-data) in conjunction with publicly [available](https://www.cms.gov/medicare/health-plans/medicareadvtgspecratestats/downloads/announcement2014.pdf) *Medicare Advantage Capitation Rates and Medicare Advantage and Part D Payment Policies and Final Call Letter* published in each year to assign the correct benchmark to each plan based on the [ratebook rate calculation data](https://www.cms.gov/medicare/payment/medicare-advantage-rates-statistics/ratebooks-supporting-data). We then construct de facto quality bonus payments based on the difference between the plan’s pre-risk adjustment benchmark and the county’s baseline benchmark, normalized by the county’s baseline benchmark, similar to the approach used in [Layton & Ryan 2015](https://onlinelibrary.wiley.com/doi/epdf/10.1111/1475-6773.12409).
- **Figure 5:** This figure presents the effective benchmark (as calculated for Figure 2), bids, and total payments.
  - Bid: We construct bids using the [plan payment data](https://www.cms.gov/medicare/health-drug-plans/plan-payment-data) and reported premiums from the [Plan Benefits Package data](https://www.cms.gov/data-research/statistics-trends-and-reports/medicare-advantagepart-d-contract-and-enrollment-data/benefits-data). Plan payment data reports risk-normalized (i.e., presented for risk score equal to one) bid government payments. For plans that bid at or below the benchmark, this value thus reflects the total bid. For the minority of plans that bid above the benchmark, the total benchmark is this plan payment data plus the Part A and B premium data from the Plan Benefits Package file.
  - Bid*RiskScore: This presents risk-adjusted bids, using the average risk score as reported in the [plan payment data](https://www.cms.gov/medicare/health-drug-plans/plan-payment-data).
  - Total Governmental Payments: We additionally use the rebate payments as reported by the [plan payment data](https://www.cms.gov/medicare/health-drug-plans/plan-payment-data) to calculate the total payments to MA plans. Note that for this amount, we do not include premiums for plans that bid above the benchmark, since that amount is paid by enrollees, not the government.
- **Figure 6:** We show the average enrollee-weighted rebate payments timeseries. Rebate amounts are reported in the [plan payment data](https://www.cms.gov/medicare/health-drug-plans/plan-payment-data).
- **Appendix Figure A.1:** This figure shows the average enrollee-weighted quartile adjustment over time. We derive quartile adjustment using the MA [ratebook rate calculation data](https://www.cms.gov/medicare/payment/medicare-advantage-rates-statistics/ratebooks-supporting-data). [Plan-level enrollment data](https://www.cms.gov/data-research/statistics-trends-and-reports/medicare-advantagepart-d-contract-and-enrollment-data) is derived from the MA landscape files.
- **Appendix Figure A.2:** This figure shows the average enrollee-weighted de facto Quality Bonus Payments in dollar terms over time. We use the star rating as reported in the [plan performance files](https://www.cms.gov/medicare/health-drug-plans/part-c-d-performance-data) in conjunction with publicly [available](https://www.cms.gov/medicare/health-plans/medicareadvtgspecratestats/downloads/announcement2014.pdf) *Medicare Advantage Capitation Rates and Medicare Advantage and Part D Payment Policies and Final Call Letter* published in each year to assign the correct benchmark to each plan based on the [ratebook rate calculation data](https://www.cms.gov/medicare/payment/medicare-advantage-rates-statistics/ratebooks-supporting-data). We then construct de facto quality bonus payments based on the difference between the plan’s pre-risk adjustment benchmark and the county’s baseline benchmark, normalized by the county’s baseline benchmark, similar to the approach used in [Layton & Ryan 2015](https://onlinelibrary.wiley.com/doi/epdf/10.1111/1475-6773.12409).
- **Appendix Figure A.3:** This figure depicts the share of total MA annual enrollment in each year with different levels of statutory quality bonus program (QBP) payments. Unlike figure 4 or appendix figure A.2, these QBP’s reflect statutory payments prior to the imposition of double-bonus payments, pre-ACA benchmark caps, or other adjustments. We assign individual plans to their QBP percentage based on the plan’s star rating per the [plan performance files](https://www.cms.gov/medicare/health-drug-plans/part-c-d-performance-data) in conjunction with publicly [available](https://www.cms.gov/medicare/health-plans/medicareadvtgspecratestats/downloads/announcement2014.pdf) *Medicare Advantage Capitation Rates and Medicare Advantage and Part D Payment Policies and Final Call Letter* published in each year, which reports the QBP percentage and rebate rate garnered by different star ratings.
- **Appendix Figure A.4:** This figure shows the share of MA enrollment in counties that are eligible for double-bonuses based on data provided in the CMS ratebook. We identify double-bonus counties using the [ratebook rate calculation data](https://www.cms.gov/medicare/payment/medicare-advantage-rates-statistics/ratebooks-supporting-data), which reports counties eligible for a double bonus in each year.
- **Appendix Figure A.5:** This figure shows the average enrollee-weighted risk score for MA plan enrollees in each year. Each plan’s risk score is reported in the [plan payment data](https://www.cms.gov/medicare/health-drug-plans/plan-payment-data). [Plan-level enrollment data](https://www.cms.gov/data-research/statistics-trends-and-reports/medicare-advantagepart-d-contract-and-enrollment-data) is derived from the MA landscape files.
- **Appendix Figure A.6:** To construct this figure, we run separate regressions for each year of county-level baseline benchmarks, benchmarks with QBP payments, and risk-adjusted benchmarks on county-level traditional Medicare spending. This figure plots the coefficient of determination or r-squared from each separate regression. We use administrative data to derive average traditional Medicare spending. Benchmarks are constructed in an identical manner to Figure 2.
- **Appendix Figure A.7:** This figure shows for each year, the average rebate rate that MA plans would face if they bid under the benchmark. We assign plans their relevant rebate rate based on the plan’s star rating per the [plan performance files](https://www.cms.gov/medicare/health-drug-plans/part-c-d-performance-data) in conjunction with publicly [available](https://www.cms.gov/medicare/health-plans/medicareadvtgspecratestats/downloads/announcement2014.pdf) *Medicare Advantage Capitation Rates and Medicare Advantage and Part D Payment Policies and Final Call Letter* published in each year, which reports the QBP percentage and rebate rate garnered by different star ratings.

**Appendix 3: Additional Results**

Appendix Figure A.1: Average Per-Enrollee Quartile Adjustment Timeseries

*Note:* This figure shows the average, enrollee-weighted, quartile benchmark percentage adjustment each year. The adjustment was introduced in 2012 (hence, in 2010 and 2011, the quartile adjustment is at 100%). In following years, baseline benchmarks are set using this this percentage so that in 2021, the average plan was adjusted to be 4.5% higher than it would have been based on traditional Medicare spending and the baseline benchmark calculation formula, absent quartile adjustments. Metrics are enrollee-weighted. This figure restricts to non-SNP, non-EGHP plans that are either HMO or local PPO plans.

Appendix Figure A.2: Average Per-Enrollee Monthly Quality Bonus Payment Timeseries

*Note:* This figure shows the average, enrollee-weighted, pre-risk adjustment monthly de-facto Quality Bonus Payment (QBP) amount in dollar terms paid to MA plans per month. The QBP was introduced in 2012. Benchmarks were increased by this QBP off of the baseline benchmark available in their area. Metrics are enrollee-weighted. This figure restricts to non-SNP, non-EGHP plans that are either HMO or local PPO.

Appendix Figure A.3: Share of Total Annual Enrollment by Statutory Quality Bonus Program Payment Percentage (i.e., not including Double-Bonus Adjustments)

*Note:* This figure depicts the share of total MA annual enrollment in each year with different levels of statutory quality bonus program (QBP) payments, prior to the imposition of double-bonus payments, pre-ACA benchmark caps, or other adjustments. Moving from left to right, the figure shows the share of enrollees in each year that have increasing statutory QBP payments. This figure restricts to non-SNP, non-EGHP plans that are either HMO or local PPO plans.

Appendix Figure A.4: Share of Total Annual Enrollment in Double-Bonus Eligible Counties

*Note:* This figure shows the share of MA enrollment in counties that are eligible for double-bonuses based on data provided in the CMS ratebook. The designation of double-bonus was introduced alongside the QBP in 2012. In double-bonus counties, benchmarks are adjusted by double the QBP. Metrics are enrollee-weighted. This figure restricts to non-SNP, non-EGHP plans that are either HMO or local PPO.

Appendix Figure A.5: Average Risk Score

*Note:* This figure shows the average risk score of enrollees in non-EGHP, non-SNP Medicare Advantage plans in each year. Metrics are enrollee-weighted. Metrics are enrollee-weighted. This figure restricts to non-SNP, non-EGHP plans that are either HMO or local PPO.

Appendix Figure A.6: R-Squared Timeseries


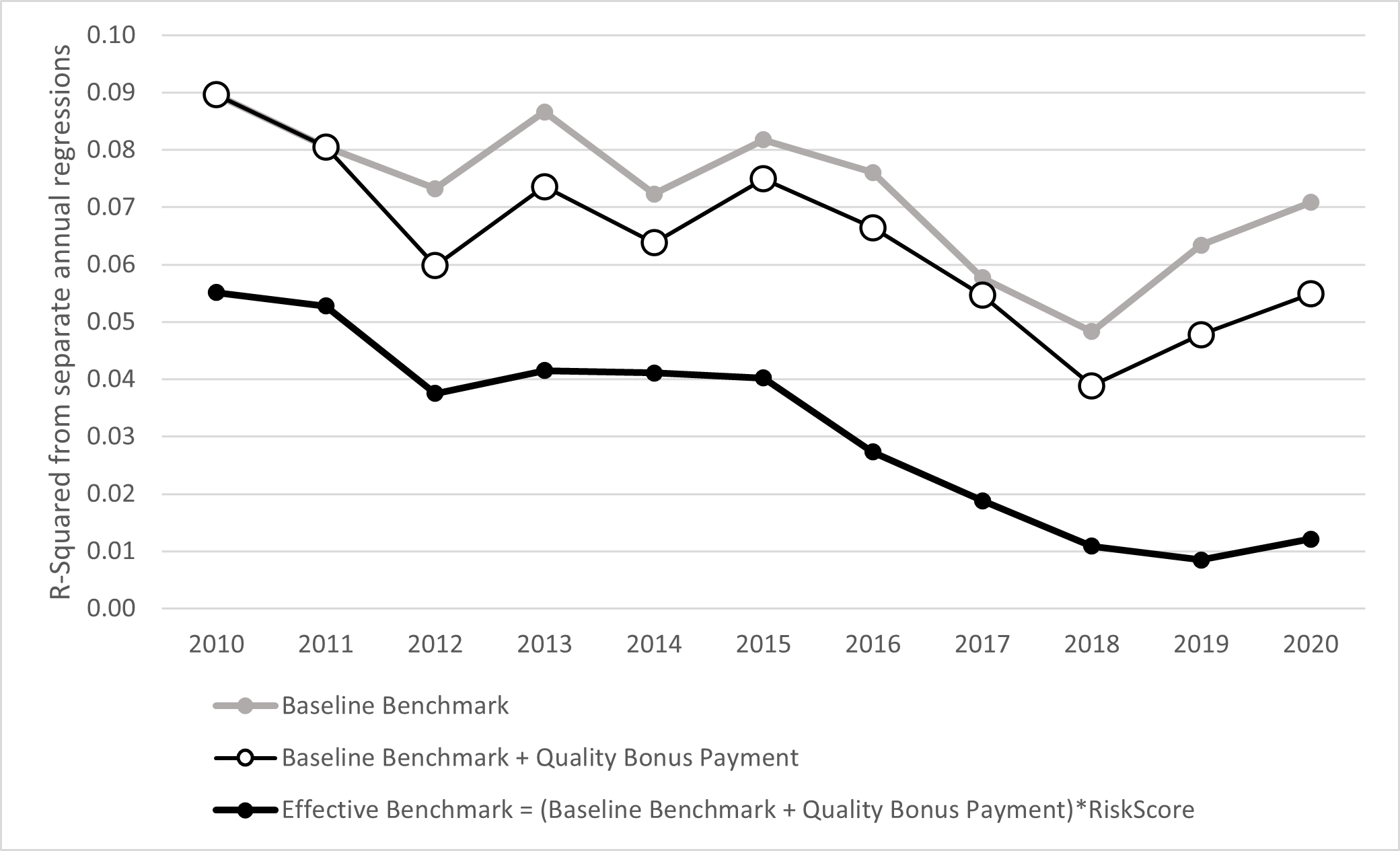


*Note:* To construct this figure, we run separate regressions for each year of county-level baseline benchmarks, benchmarks with QBP payments, and risk-adjusted benchmarks on county-level traditional Medicare spending. This figure plots the coefficient of determination or r-squared from each separate regression. Metrics are enrollee-weighted. This figure restricts to non-SNP, non-EGHP plans that are either HMO or local PPO.

Appendix Figure A.7: Average Rebate Rate

*Note:* This figure shows for each year, the average rebate rate that Medicare Advantage plans would face if they bid under the benchmark. Metrics are enrollee-weighted. Metrics are enrollee-weighted. This figure restricts to non-SNP, non-EGHP plans that are either HMO or local PPO plans.
