# Supplementary material for: Growing divergence between Medicare Advantage plan bids and payments to plans
Source: Health Aff Sch. 2024 Aug 5;2(8):qxae093. doi: 10.1093/haschl/qxae093 (PMC11342956; doi:10.1093/haschl/qxae093)
Supplement: qxae093_Supplementary_Data [file qxae093_supplementary_data.zip › ICMJE coi_disclosure Trish MA Payment.docx]

| ICMJE DISCLOSURE FORM | |
| --- | --- |
| **Date:** | 4/29/2024 |
| **Your Name:** | Erin Trish |
| **Manuscript Title:** | Comparing Expected Out-of-Pocket Costs in Medicare Advantage and Fee for Service Medicare, 2014-2020 |
| **Manuscript Number (if known):** | Growing Divergence Between Medicare Advantage Plan Bids and Payments to Plans |
| In the interest of transparency, we ask you to disclose all relationships/activities/interests listed below that are related to the content of your manuscript. “Related” means any relation with for-profit or not-for-profit third parties whose interests may be affected by the content of the manuscript. Disclosure represents a commitment to transparency and does not necessarily indicate a bias. If you are in doubt about whether to list a relationship/activity/interest, it is preferable that you do so.  The author’s relationships/activities/interests should be defined broadly. For example, if your manuscript pertains to the epidemiology of hypertension, you should declare all relationships with manufacturers of antihypertensive medication, even if that medication is not mentioned in the manuscript.  In item #1 below, report all support for the work reported in this manuscript without time limit. For all other items, the time frame for disclosure is the past 36 months. | |

|  | | | **Name all entities with whom you have this relationship or indicate none (add rows as needed)** | **Specifications/Comments (e.g., if payments were made to you or to your institution)** |
| --- | --- | --- | --- | --- |
| **Time frame: Since the initial planning of the work** | | | | |
| **1** | All support for the present manuscript (e.g., funding, provision of study materials, medical writing, article processing charges, etc.)  **No time limit for this item.** | | \|  \| **None** \| \| --- \| --- \|  \| Arnold Ventures \|  \| \| --- \| --- \| \|  \|  \| \|  \| Click the tab key to add additional rows. \| | |
| **Time frame: past 36 months** | | | | |
| **2** | | Grants or contracts from any entity (if not indicated in item #1 above). | \|  \| **None** \| \| --- \| --- \|  \| Alexion \| All were provided to my institution \| \| --- \| --- \| \| Amgen \|  \| \| Arnold Ventures \|  \| \| Biogen \|  \| \| Biomarin \|  \| \| Blue Cross Blue Shield of Arizona \|  \| \| Blue Cross Blue Shield of Massachusetts \|  \| \| Bristol Myers Squibb \|  \| \| California Hospital Association \|  \| \| Cedars Sinai Helath System \|  \| \| Charles Koch Foundation \|  \| \| Commonspirit \|  \| \| Commonwealth Fund \|  \| \| Edwards Lifesciences \|  \| \| Eli Lilly \|  \| \| Gates Ventures \|  \| \| Genentech \|  \| \| Gilead Sciences \|  \| \| GRAIL \|  \| \| IVI Foundation \|  \| \| Johnson & Johnson \|  \| \| Kaiser Family Foundation \|  \| \| National Institutes of Health \|  \| \| Novartis \|  \| \| Pfizer \|  \| \| RA Capital \|  \| \| Roche \|  \| | |
| **3** | | Royalties or licenses | \|  \| **None** \| \| --- \| --- \|  \|  \|  \| \| --- \| --- \| \|  \|  \| \|  \|  \| | |
| **4** | | Consulting fees | \|  \| **None** \| \| --- \| --- \|  \| Quant Health \|  \| \| --- \| --- \| \|  \|  \| \|  \|  \| \|  \|  \| | |
| **5** | | Payment or honoraria for lectures, presentations, speakers bureaus, manuscript writing or educational events | \|  \| **None** \| \| --- \| --- \|  \| Cedars Sinai Health System \|  \| \| --- \| --- \| \|  \|  \| \|  \|  \| | |
| **6** | | Payment for expert testimony | \|  \| **None** \| \| --- \| --- \|  \| Centene \|  \| \| --- \| --- \| \| Cornerstone Research \|  \| \| Guardian Pharmacy \|  \| \| Mallinckrodt \|  \| \| Varian Medical Systems \|  \| | |
| **7** | | Support for attending meetings and/or travel | \|  \| **None** \| \| --- \| --- \|  \|  \|  \| \| --- \| --- \| \|  \|  \| \|  \|  \| | |
| **8** | | Patents planned, issued or pending | \|  \| **None** \| \| --- \| --- \|  \|  \|  \| \| --- \| --- \| \|  \|  \| \|  \|  \| | |
| **9** | | Participation on a Data Safety Monitoring Board or Advisory Board | \|  \| **None** \| \| --- \| --- \|  \|  \|  \| \| --- \| --- \| \|  \|  \| \|  \|  \| | |
| **10** | | Leadership or fiduciary role in other board, society, committee or advocacy group, paid or unpaid | \|  \| **None** \| \| --- \| --- \|  \| American Journal of Managed Care \| Editorial Board \| \| --- \| --- \| \| Medical Care Research and Review \| Editorial Board \| \|  \|  \| | |
| **11** | | Stock or stock options | \|  \| **None** \| \| --- \| --- \|  \|  \|  \| \| --- \| --- \| \|  \|  \| \|  \|  \| | |
| **12** | | Receipt of equipment, materials, drugs, medical writing, gifts or other services | \|  \| **None** \| \| --- \| --- \|  \|  \|  \| \| --- \| --- \| \|  \|  \| \|  \|  \| | |
| **13** | | Other financial or non-financial interests | \|  \| **None** \| \| --- \| --- \|  \|  \|  \| \| --- \| --- \| \|  \|  \| \|  \|  \| | |
|  | |  |  | |
| **Please place an “X” next to the following statement to indicate your agreement:** | | | | |
|  | | I certify that I have answered every question and have not altered the wording of any of the questions on this form. | | |
